# Relationship between Problematic Internet Use, Sleep Problems, and Oral Health in Korean Adolescents: A National Survey

**DOI:** 10.3390/ijerph15091870

**Published:** 2018-08-29

**Authors:** Kyung-Yi Do, Kang-Sook Lee

**Affiliations:** 1 Department of Public Health, Graduate School, The Catholic University of Korea, 222 Banpo-daero, Seocho-gu, Seoul 06591, Korea; dkl8684@naver.com; 2 Department of Preventive Medicine, College of Medicine, The Catholic University of Korea, 222 Banpo-daero, Seocho-gu, Seoul 06591, Korea

**Keywords:** adolescents, problematic internet use, oral health, sleep, Korea, Korean youth risk behavior web-based survey

## Abstract

We examined the relationship between Problematic Internet Use (PIU), sleep (sleep satisfaction, sleep duration), and experience of oral disease symptoms in Korean adolescents by gender. This cross-sectional study utilized the 6th (2010) Korean Youth Risk Behavior Web-based Survey. Participants comprised 74,980 students from 400 middle schools and 400 high schools nationwide. Among these, 73,238 students from 799 schools (38,391 boys, 34,847 girls, aged 13–18 years) were included in the analysis (inclusion rate = 97.7%). Multiple logistic regression and analysis of moment structures (AMOS) analyses were performed to identify meaningful relationships between the three factors. The “high risk group” of problematic internet usage had increased experience of oral disease symptoms (boys: adjusted odds ratio (AOR) = 1.92, 95% confidence interval (CI) = 1.63–2.28, girls: AOR = 1.98, 95% CI = 1.50–2.63) compared to the general group. Boys who used the Internet for “5–6 h” had a higher risk of oral disease symptoms compared to those who used it for “less than 1 h” (OR = 1.24, 95% CI = 1.01–1.53); however, this difference was not significant in Models II and III. For girls, the risk of 5–6 h of use (Model I: OR = 1.69, 95% CI = 1.40–2.04) was higher than that of the boys. In addition, the difference was significant in Models II and III for girl students who used the Internet for 5–6 h. In subgroup analysis, the high-risk group had a higher odds ratio for mild symptoms of bad breath to severe symptoms such as sore and bleeding gums. In addition, in the path analysis, PIU affected sleep and indirectly affected oral health. Direct and indirect causal relationships between the three factors were confirmed. Therefore, it is important to recognize that PIU can have a detrimental effect on mental, physical, and oral health.

## 1. Introduction

In modern society, Internet use has become an indispensable element of everyday life, bringing conveniences from around the world [1,2], while excessive use of the Internet has caused serious social problems such as “Internet addiction” [3]. Korea has the highest proportion of Internet users in the world, and its use has increased dramatically due to the recent surge of smartphones, PC tablets, and other mobile devices [1,2]. Conversely, excessive Internet use has sparked serious social problems such as Internet addiction.

Adolescence is a transitional period from childhood to adulthood. It is a period of intense development, both physically and mentally, and is a very important period for the formation of health habits that can affect one’s whole life [4]. Adolescents lack self-control abilities because of psychological immaturity, are more susceptible to addiction than adults because of their brimming curiosity and have more difficulty in recognizing the adverse effects of Internet addiction [5]. Moreover, excessive internet use such as playing games, shopping, chatting, watching pornography, and so on, is a cause of reduced physical activity and sleep time, which has adverse effects on physical and mental health during adolescence, when one should be forming proper health habits [3,6,7].

Previous studies have suggested that problematic Internet use (PIU) can cause psychosocial problems, such as adolescent sleep deprivation [2], adverse effects on mental health including excessive stress, depression, anxiety, lack of self-esteem, and suicidal ideation [8,9,10,11]. In addition, An et al. reported that the higher the PIU score, the higher the risk ratio of physical symptoms and psychological symptoms [11]. Lam’s systematic review presented a conceptual model of the relationship between Internet addiction and depression, in which Internet addiction influences neurological changes and sleep problems, causing anxiety and stress, and eventually depression. In the final review, all PIU was associated with sleep problems (reduced sleep time, poor sleep quality, insomnia, etc.) [12]. Previous studies have suggested the following mechanisms that can be explained in relation to problematic Internet use and sleep: (1) Media use replaces sleep, (2) Media use increases arousal problems, and (3) Bright light exposure delays the circadian rhythm [13,14]. Therefore, if a long time is spent at night on the internet, this will make it harder for an individual to fall asleep and will cause irregular sleeping habits. This, in turn, can increase daytime sleepiness and fatigue, which will interfere with school life [15,16]. According to these prior studies, PIU may cause sleep deprivation. If sleep deprivation continues, the immune system will eventually become vulnerable to infection, and indirectly, oral health behaviors will be hindered, leading to oral diseases.

According to previous studies, sleep deprivation is closely associated with oral diseases such as chronic periodontitis [17,18,19,20], as lack of sleep could cause vulnerability to infection due to decreased immunity, excessive fatigue, and so on. This in turn could lead to oral diseases including chronic inflammatory periodontal diseases [17]. In a recent study, subjects with a sleep time of less than six hours reported a 1.25-fold increased risk of gingival inflammation [21]. In addition, PIU can affect the health behaviors of adolescents. Celik et al. reported that problematic Internet use affected eating times and amounts due to the long-term occupation with the Internet. The associated frequent snacking could lead to poor dietary attitudes, such as skipping meals [22]. Poor eating habits will also affect oral health behaviors, such as regular eating and the accompanying regular brushing [2,23]. In a recent study, Do and Lee reported that the high-risk group for excessive Internet use (EIU) reported that the risk of less daily brushing had an odds ratio (OR) = 4.04 times higher than that of the general group, and their risk of not brushing after lunch had an OR = 1.7. Further, it was shown that EIU can decrease adolescents’ oral health behaviors, which may adversely affect oral health if it lasts for a long time [23].

Around the world, 60–90% of children and adolescents experience severe pain and discomfort because of tooth decay. Gingivitis is common in children and adolescents, and severe progressive periodontitis has been reported [24]. Because periodontitis increases rapidly during adolescence, if oral hygiene is neglected during this period or if periodontal disease is not treated early, severe oral pain and difficulty in chewing may cause discomfort during ingestion of food and can also interfere with school life [25]. Adolescent oral health problems may be regarded as less serious in the short term in comparison to adults, but the long-term effects of poor oral health problems can be as severe for adults [26]. Therefore, more attention is needed, and it is necessary to identify new risk factors other than those that have been found previously to have a detrimental effect on the oral health and intervene accordingly. However, most of the research related to PIU has mostly focused on mental health related issues [8,12,27,28,29,30]. Recently, research has been carried out on the relationship between neuropsychiatric symptoms, psychosocial symptoms, and physical health [9,11], but the relationship between PIU and oral health has not yet been studied. The relationship between PIU and problems with sleep [2,13,14,15] and oral diseases have been proved through various studies [17,18,19,20,21]. Korean adolescents’ smartphone addiction rates increased steadily from 11.4% in 2011 to 31.6% in 2015, but there was a slight decline to 30.3% in 2017 [31]. This is still a serious health problem among Korean youth and is a health risk factor that requires intervention. Therefore, it is of great importance to study the relationship between PIU, sleep problems, and oral health based on the abovementioned studies. This study suggests that PIU is a new risk factor that causes problems such as sleep deprivation and eventually deleterious effects on oral health, and that it is necessary for related scientific research to be actively carried out.

**Figure 1:**
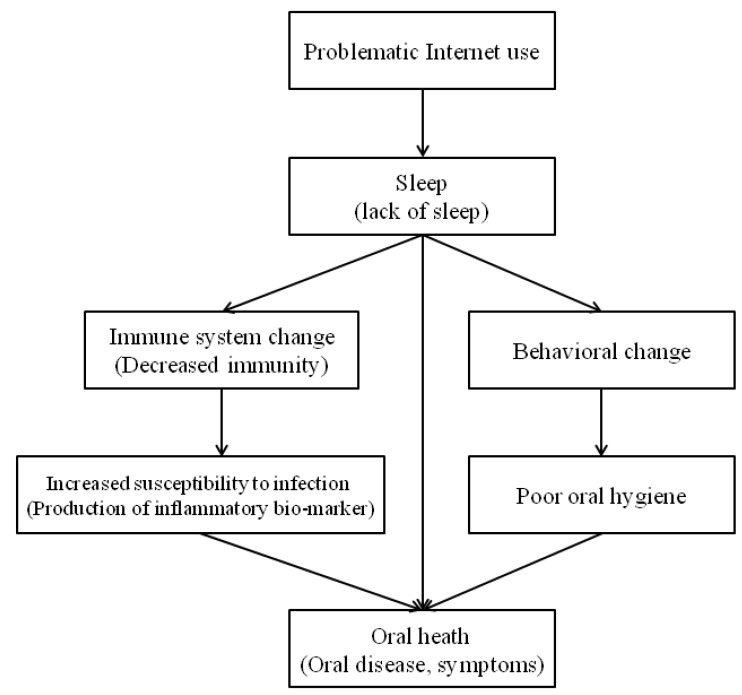
A conceptual model for explanatory relationship between problematic Internet use, sleep, and oral health [12,17,19,32].

The hypotheses of this study are: (1) PIU affects sleep deprivation and indirectly affects oral health (e.g., oral disease symptoms) through mediation. (2) PIU will be directly related to oral health. The aim of the study was to investigate the relationship between PIU, sleep (sleep satisfaction, sleep duration), and experience of oral disease symptoms using national data representative of Korean adolescents by gender. A theoretical conceptual model for explaining the relevance of these three variables was developed (Figure 1).

## 2. Materials and Methods

### 2.1. Participants

This study was based on the sixth edition of the Korean youth risk behavior web-based survey (KYRBS), which was conducted from 1 September 2010 to 24 October 2010. It used a complex sample design to obtain a sample representative of middle school and high school students nationwide. The study population was students (as of April 2009) of middle schools and high schools nationwide, and the sampling process was divided into the stages of population stratification, sample distribution, and sampling. In the population stratification stage, we used 45 local districts (*gun*) and grade level (middle school, general high school, and specialized high school) as the stratification variables and divided the population into 135 layers. Stratified cluster sampling was used for sampling, where the primary extraction unit was the school and the secondary extraction unit was the grade. All students in the grade selected as the sample were surveyed, and long-term absentees, special children, and students with literacy disabilities were excluded from the sample. The KYRBS did not collect any personal information from the participants (e.g., names, home addresses, phone numbers, school names, or social security numbers). Written informed consent was not obtained from the participants. The participants of this study comprised 74,980 students from 400 middle schools and 400 high schools nationwide. The final participation rate was 97.7% and included 799 schools and 73,238 students (38,391 boys, 34,847 girls, age range = 13–18 years). The study was approved by the Institutional Review Board of the Catholic University of Korea (MC16EISI0083).

### 2.2. Measurements

The KYRBS is an anonymous, self-reported online survey that has been annually conducted by the Ministry of Education, the Ministry of Health and Welfare, and Centers for Disease Control and Prevention since 2005. It targets all Korean middle school and high school students to determine their current health status and health behavioral trends. The survey includes 128 questions in 14 areas: health behaviors, oral health, mental health, Internet addiction, smoking, drinking, obesity, physical activity, eating habits, etc. This study extracted and used questions related to oral health (oral symptoms, six factors), Internet addiction (Korean version of Internet Addiction Proneness Self-Short Form, KS-scale, 20 items, including Internet use duration on weekends for purposes other than studying), general characteristics (five factors), and health related factors (five factors) from the KYRBS.

### 2.3. General Demographic Characteristics

We examined sex, grade, perceived family economic status, academic performance, and living status in this study. Grade was categorized into middle school: 1st/2nd/3rd grade, and high school: 1st/2nd/3rd grade. Perceived family economic status was re-categorized from “high, mid-high, middle, mid-low, and low” to “high, middle, and low”, and academic performance was also re-categorized from “high, mid-high, middle, mid-low, and low” to “high, middle, and low”. Living status was categorized into “living with family, living with relatives, boarding, living in dorm (including living with friends), and living in a care facility”.

### 2.4. Health Related Factors

Health related factors comprised alcohol and smoking experience, stress level, perceived sleep satisfaction, and sleep duration. Alcohol experience was determined by “*yes* or *no*” responses to the question, “Have you ever had more than one glass of alcohol?” Smoking experience was determined by “*yes* or *no*” responses to the question, “Have you ever inhaled from a cigarette even once or twice?” Stress level was determined by responses comprising “*very much*, *a lot*, *a little*, *not much*, and *not at all*” to the question, “In general, how much stress do you feel?” For sleep satisfaction, the question asked was, “Was the amount of sleep you had in the past 7 days sufficient for relieving fatigue?” Responses were categorized as “*completely sufficient*, *sufficient*, *moderate*, *not sufficient*, and *not at all sufficient*” and used as nominal variables. The responses “*not at all sufficient*” to “*completely sufficient*” were converted into scores ranging from 1 to 5 and used as continuous variables for the analysis of moment structures (AMOS). Sleep duration was assessed by asking participants “In the past 7 days, what time did you usually sleep in bed and wake up in the morning?” The participants answered this question directly in the survey. Their responses were calculated and used for the duration of sleep. For Chi-square analysis, we categorized sleep duration as “within 5 h 5–7 h, 7–9 h, 9 h or more” and these were used as continuous variables in logistic regression analysis and AMOS path analysis.

### 2.5. Problematic Internet Use

To measure PIU, we used the KS-scale, which was developed by the National Information Society Agency [33]. The KS scale is a measurement tool used for diagnosing adolescent internet addiction and is a self-report survey that consists of six sub-factors and 20 questions. Each question was measured on a 4-point Likert scale (1 = *never*, 2 = *sometimes*, 3 = *often*, and 4 = *nearly always*), and provides a score of 20 to 80 points. The six sub-factors consisted of the following: (1) disturbance of adaptive functions (six questions), (2) addictive automatic thoughts (one question), (3) withdrawal (four questions), (4) virtual interpersonal relationships (three questions), (5) deviant behavior (two questions), and (6) tolerance (four questions). The reliability coefficient of the measurement tool was highly satisfactory (Cronbach’s α = 0.91), and this study distinguished PIU into three groups: high-risk group, potential-risk group, and general group according to participants’ KS-scale score. Those who scored 53 or higher on the KS-scale were defined as the “high-risk group,” those who scored between 48–52 were deemed the “potential risk group,” and the remaining were defined as the “general group” (classification was based on the Korea National Information Society Agency [33]; Park et al. [28]). In the AMOS analysis, we used PIU (KS-scale) as a continuous variable. In addition, Internet use on the weekends for purposes other than studying was evaluated on the basis of self-recorded internet usage duration (i.e., less than 1 h, ≥1–2 h, ≥2–3 h, ≥3–4 h, ≥4–5 h, ≥5–6 h, and more than 6 h).

### 2.6. Oral Health

For oral health, we used six symptoms used by the World Health Organization as indicators of oral health for international comparison [34]. We asked respondents to answer “*yes* or *no*” to whether they had experienced the following symptoms in the past 12 months: “chipped or broken tooth, toothache when eating, throbbing and sore tooth, sore and bleeding gums, pain in tongue or inside the cheeks, and bad breath”. A logistic regression analysis was performed to determine the severity of each symptom according to PIU. For AMOS analysis, each symptom was given a score of 1 for *yes* and 0 for *no*, and the scores for the six symptoms were summed for a total score ranging from 0 to 6, which was used as a continuous variable. In addition, oral symptoms were divided into two bipartite for chi-square (χ^2^) analysis and logistic regression analysis. A total score ranging from 0 to 6, the scores were 0 for “no oral symptoms experienced (*no*)” and 1–6 points for “oral symptoms experienced (*yes*)”.

### 2.7. Statistical Analysis

The study data were analyzed using a complex sample analysis module, considering the stratification variables, cluster variables, and weight. First we applied a Rao-Scott χ^2^-test to present *N* (weight %) to examine whether there was a significant difference in the distribution of oral disease symptoms experienced according to the participants’ general characteristics, health related factors, and PIU. Next, we performed a complex sample logistic regression analysis and presented the OR and 95% CI to identify the relationship between PIU, oral disease symptoms, and sleep problems (sleep satisfaction, sleep duration). Model I investigated the relationship between PIU and oral disease symptoms (unadjusted). Model II investigated the relationship between PIU, oral disease symptoms, and sleep problems (unadjusted). Model III investigated the relationship between PIU, oral disease symptoms, and sleep after adjusting for covariates (sex, grade, academic performance, perceived family economic status, living status, alcohol, smoking, and stress level). As a subgroup analysis, we then conducted logistic regression analysis to determine the severity of each symptom according to PIU. Finally, we conducted AMOS structural equation modeling to examine the direct and indirect causation of the three factors of PIU, sleep, and oral disease symptoms. Statistical analyses were conducted using PASW 18.0 Version (SPSS Inc., Chicago, IL, USA) and AMOS (version 24.0; SPSS Inc.). Statistical significance was verified at the *p* < 0.05 level.

## 3. Results

### 3.1. Oral Disease Symptoms Experienced According to General Characteristics

More female students experienced oral disease symptoms than male students did, and more high school students experienced oral disease symptoms than middle school students did. Students with low academic performance experienced oral disease symptoms significantly more than students with high academic performance did. Students with a low perceived family economic status had significantly higher oral disease symptoms than the high group did. Students living with relatives had the highest rate of oral disease symptoms experienced, whereas students living in a care facility had the lowest (see Table 1).

### 3.2. Oral Disease Symptoms Experienced According to Health Related Factors and Problematic Internet Use

Students who had used alcohol and cigarettes had a higher rate of oral disease symptoms than those who had not. For stress level, students who felt “*very much*” stressed had a much higher rate of oral disease symptoms than students who felt “*not at all stressed*”. Regarding sleep satisfaction, students who answered “*not at all sufficient*” had a much higher rate of oral disease symptoms than those who answered “*completely sufficient*”. For sleep duration, subjects with a sleep time of less than 5 h had the highest oral disease symptoms, but this was not statistically significant. For PIU, the potential-risk group and high-risk group had higher rates of oral disease symptoms than the general group did. In addition, regarding weekend Internet usage time for purposes other than learning, students with 1–2 h of use had lower rates of oral disease symptoms than students with less than 1 h of use. Moreover, concerning usage time of more than 2 h, the rate of oral symptom experience increased as usage time increased. The oral symptom experience rate was highest among students who reported using the internet for 5–6 h (73.4%; see Table 2).

### 3.3. Risk of Oral Disease Symptoms According to Problematic Internet Use and Sleep Problems by Gender

For boys, the risk of oral disease symptoms increased in the “high risk group” in Models I and II, and it slightly decreased following the adjustment in Model III (after adjusting all covariates); however, there was still a considerable risk (boys: AOR = 1.92, girls: AOR = 1.98). In terms of Internet usage, the risk of oral disease symptoms increased as the usage time increased. Boys who used the Internet for 5–6 h had the greatest risk of oral health problems among all Internet usage groups (Model I, OR = 1.24, 95% CI =1.01–1.53). However, there were no significant differences in Models II and III. For girls, the risk of oral disease symptoms increased as the usage time increased; the same pattern was observed in boys. The risk among Internet users of 5–6 h (Model I, OR = 1.69, 95% CI = 1.40–2.04) was higher than that of the boys (OR = 1.24). In addition, the differences were significant in Model II (AOR = 1.61, 95% CI = 1.33–1.94) and Model III (AOR = 1.38, 95% CI = 1.14–1.67) for girls who used the internet for 5–6 h. Concerning sleep satisfaction, for all students (boys and girls), the risk of oral disease symptoms increased for students who answered “*not at all sufficient*” compared to those who answered *“completely sufficient*.” Sleep duration was not statistically significant (see Table 3).

### 3.4. Subgroup Analysis of the Relationship between Problematic Internet Use and Each of the Six Oral Disease Symptoms

The risk of experiencing symptoms of six oral diseases was higher in the potential risk group and the high-risk group than in the general group. In particular, in the high-risk group, “bad breath” had an OR 2.66 times (95% CI: 2.407–2.930) higher than the general group. For “pain in tongue or inside cheeks,” the OR was 2.29 times (95% CI: 2.052–2.548) higher and for “sore and bleeding gum,” the OR was 2.11 times (1.905–2.331) higher. The remaining severe symptoms were 1.5 times higher in the high-risk group than in the general group. After all the covariates were adjusted, there was an increased risk of five oral symptoms except for “chipped or broken teeth” and a slight decrease in AOR, but there was still a high risk of experiencing oral disease symptoms in the potential risk group and the high-risk group (see Table 4).

### 3.5. Path Analysis for Direct and Indirect Causal Relationships between Problematic Internet Use, Sleep, and Oral Health

**Figure 2:**
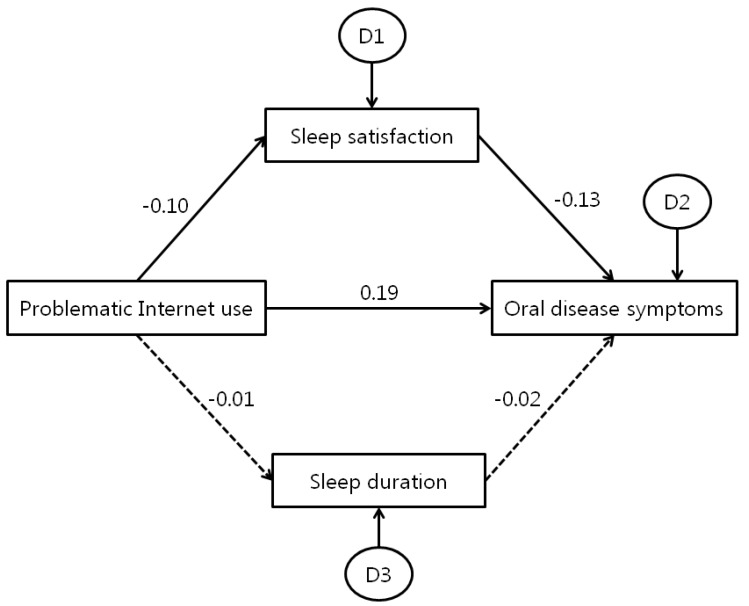
Path diagram showing the casual relationships between problematic internet use, sleep, and oral disease symptoms. All values are standardized regression weights.

According to the AMOS analysis results, PIU significantly predicted oral disease symptoms (Standardized Regression Weights, β = 0.186; Critical Ratio, C.R = 51.664; *p* < 0.001). Sleep satisfaction significantly predicted oral disease symptoms (β = −0.126; C.R. = −34.873; *p* < 0.001). Low sleep satisfaction significantly predicted more oral disease symptoms. Sleep satisfaction partially mediated PIU and oral disease symptoms (indirect effect β = 0.012). Therefore, PIU not only directly affected oral health, but also sleep satisfaction and indirectly affected oral health (total effect B = 0.199). Moreover, PIU lowered sleep duration (β = −0.01; C.R. = −2.766; *p* = 0.006) and sleep duration significantly predicted oral disease symptoms (β = −0.015; C.R. = −4.269; *p* = 0.001); however, the relationship between PIU and oral health was not indirectly affected by the mediating effects of sleep duration (Figure 2).

## 4. Discussion

We examined the relationship between PIU, sleep (sleep satisfaction, sleep duration), and experience of oral disease symptoms in Korean adolescents by gender. Our main finding was that PIU (KS-scale) increased the rate of oral disease symptoms by 1.92 times for boys and 1.98 times for girls (Model III). PIU was associated with a higher risk of experiencing symptoms of 6 oral diseases in the potential group and the high-risk group than in the general group.

According to a 2014 report, Korean adolescents’ Internet addiction rate reached serious levels (12.5%). The smartphone addiction rate for 10–19-year olds is reported to be 30.3% [31], and it has been steadily increasing over the past several years. Moreover, this pattern is likely to continue. Further, 23.8% of adolescents experienced interference in their academics and work due to excessive mobile messenger use; therefore, attention to and designing interventions for Korean adolescents’ internet addiction are desperately needed measures [35]. Internet addiction is a kind of behavior-management spectrum disorder where one experiences withdrawal and tolerance toward using the Internet due to excessive use. The inability to self-regulate one’s Internet use can negatively affect one’s life and cause problems pertaining to school, home, work, health, and personal relationships [3,35,36]. The American Psychiatric Association recently added “Internet Gaming Disorder” to Section III of the Diagnostic and Statistical Manual of Mental Disorders fifth edition and stipulated computer and Internet addiction as a sub-category of behavioral addiction in the new edition of the International Classification of Diseases [8,37]. Internet addiction has begun to be recognized as a health problem not only in South Korea, but also worldwide [38]; however, due to its novelty as a disorder, more research is necessary.

Previous studies have reported that PIU is associated with mental health problems such as increased stress, depression, anxiety, and lack of sleep [7,10,30]. Excessive Internet use may lead to mental health problems such as stress and depression, which may ultimately lead to poor oral hygiene by reducing oral health behaviors [3,39,40,41]. Recently, Park et al. [7] reported that subjects with depressive symptoms had a 1.3 times lower daily tooth brushing frequency, 1.4 times more periodontal bleeding, and experienced more toothache and temporomandibular disorder pain. However, the relationship between mental health and oral health remains unclear and controversial. Therefore, longitudinal studies should be conducted to demonstrate clear causal relationships, and the scientific evidence for this field should be further strengthened by looking for the most recent and best evidence through systematic review studies [42].

In the present study, for sleep satisfaction, risk of oral disease symptoms increased for students who answered “*not at all sufficient*” (boys: AOR = 1.40, girls: AOR = 1.44, Model III) compared to those who answered “*completely sufficient*.” Problematic internet use is closely related to sleep deprivation; one possible explanation is that excessive Internet users find it difficult to fall asleep and even more difficult to fall into deep sleep, which leads to a vicious cycle, eventually resulting in Internet addiction [15]. Excessive use of electronic media such as smart phones and desktop computers can delay going to bed to sleep and reduce sleep duration. In addition, bright light on the screen suppresses melatonin secretion, causes phase delay in the biological clock, and increases mental and physiological arousal to reduce total sleep time [2]. Sleep is essential for the maintenance of physical and mental health as well as for the functioning of daily life [6,18]. However, according to a recent report, the average hours of sleep on a weekday for adolescents in Korea was lower than the 8.5–9.25 h (not including naps) recommended by the National Sleep Foundation for people aged 10–17 years [43]. Choi et al. targeted 2336 high school students and examined the relationship between excessive Internet use and excessive daytime sleepiness (EDS). Choi et al. found that the Internet addiction group (37.7%) had a 30.3% higher risk of EDS than the control group did (7.4%) [16]. Another study reported that internet addiction reduces hours of sleep during night time and is related to poor quality sleep and insomnia. Fewer than four hours of nighttime sleep increases the secretion of cortisol, a stress-increasing hormone, and interleukin-1 (IL-1), IL-6, and IL-7 a type of cytokine that controls immune functions and tumor necrosis factor alpha (TNF-α) to beyond necessary levels [44,45,46]. This could cause damage to bones, tissue, and cardiovascular tissue [46]. Moreover, dull oral health behaviors could occur due to excessive fatigue, thereby accumulating plaque, and resulting in poor oral hygiene, eventually inducing oral diseases such as periodontitis [17,18,47,48].

In path analysis, PIU directly affected sleep and indirectly affected oral health. Direct and Indirect causal relationships between the three factors were also confirmed. PIU significantly predicted oral disease symptoms. Sleep satisfaction significantly predicted oral disease symptoms, and sleep satisfaction partially mediated PIU and oral disease symptoms. Therefore, PIU not only directly affected oral health, but also affected sleep satisfaction and indirectly affected oral health. Moreover, PIU lowered sleep duration, and sleep duration significantly predicted oral disease symptoms. Thus, as mentioned above, our findings were supported in that scientific evidence was found for how PIU can adversely affect sleeping habits, as well as the medical mechanisms involved in how sleep deprivation can have a deleterious effect on oral health. However, few studies have shown that problematic Internet use can directly affect oral health behaviors and oral disease symptoms. There is still no scientific basis to fully explain the relationship between these two factors. Therefore, we must pay attention to their interpretation. Studies should be conducted to prove a clear causal relationship between these two factors, and active research in this field needs to continue.

In this study, students who used the Internet for more than six hours and 5–6 h had a higher risk of negative oral symptoms than students who used the Internet for less than one hour. In addition, this study’s interesting results are that the risk of oral disease symptoms increased as the time spent on Internet usage increased, but the odds ratio for girls was higher than that for boys. After adjusting for the covariates, the differences among the boys were not significant, while those for the girl students were still significantly higher at “5–6 h” and “more than six hours”. According to a recent study, the rate of Internet use among girls is increasing more rapidly than that among boys, and girls are more likely to use it for social networking services (SNS), Face book, shopping, games, information acquisition, and formation of relationships in the virtual world. This is a different pattern than can be seen from the boys’ main use of the Internet, which is for gaming purposes [23,28]. Therefore, the Internet addiction path may be different between boys and girls, and the effect of Internet addiction on health status will also be different. Therefore, there is a need for more detailed research. However, conversely, the risk of negative oral symptoms was actually lower in students with 1–2 h of Internet use. A recent study by Park [49] reported that people who use the Internet for an appropriate amount of time (i.e., an average of 1 to 3 h per day) reported being less stressed and had higher levels of happiness than those who do not use the Internet at all. Therefore, Park reported a positive side of appropriate Internet usage whereas many studies have only emphasized its negative aspects [49]. As such, though Internet usage time is the most powerful predictor of Internet addiction, there is no clear scientific evidence that suggests specific standards of excessive Internet usage and appropriate Internet usage time as of yet. The reasons are presumed to be differences in research methods and participants among existing research, different purposes of use by sex and age, different standards and measurement tools of Internet addiction predictors, different Internet addiction rates by country, and more [39,50].

### Limitations and Strengths

First, being a cross-sectional study, this study is limited in that it cannot state a direct causal relationship and a temporal relationship between PIU and oral health. Therefore, epidemiological and longitudinal research should be implemented to identify a direct causal relationship and possible medical association between PIU and oral health. Second, in this study, all variables were composed of self-report questions; therefore, reporting bias may exist because adolescents can underreport their Internet usage time. Third, there was a lack of validity in assessing sleep problems, as the sleep-related variable was measured with two items (sleep satisfaction, sleep duration). In further studies, it will be necessary to accurately assess the subjects’ sleep problems using reliable and valid measurement tools. Fourth, the fact that the dependent variable of oral health did not include an actual oral examination diagnosis by experts and that we were unable to adjust oral health behaviors such as daily frequency of brushing teeth, which has an impact on oral health, could be considered further limitations. Fifth, we started this study in 2016 and completed it in 2017. However, we used 2010 data without using the most recent data for 2016. This is because score on the KS-scale, a tool measure Internet addiction was used as the independent variable in this study. KS-scale is an Internet addiction measurement tool that had not been investigated by the end of the 2010 survey. Since 2011, Internet usage patterns have been surveyed only on weekdays and weekends, except for learning purposes (entertainment, games, SNS, et al.). As a measure of Internet addiction, the KS-scale index is more reliable and valid. This study was cross-sectional and has limitations that do not reflect its timely appropriateness; however, the problem of Internet addiction is a health problem for adolescents that has not changed since the past till the present. Finally, there is a lack of related evidence as there have been very few studies on PIU and oral health as of yet. Therefore, research to support this study should be actively conducted using well-designed research methodology and the use of valid approaches for the measurement of the independent and dependent variables.

Despite these limitations, this study is meaningful in that it is the first to identify the relationships and risks of PIU, sleep, and oral health based on the KYRBS, a government-approved statistical dataset that is representative of Korean adolescents. It also has a great advantage in that the findings can be generalized to all Korean adolescents. Moreover, the factor of Internet usage time lacks validity in diagnosing Internet addiction. On the other hand, this study is meaningful in that it derived more reliable results than existing studies by using the Korean version of the KS-scale in the analysis. This is beneficial because it has confirmed reliability and validity as a predictor of adolescent Internet addiction and it includes Internet use duration on weekends for purposes other than studying, which is a strong predictor of Internet addiction [33].

## 5. Conclusions

We confirmed that PIU directly affects sleep and indirectly affects oral health, as well as confirming direct and indirect causal relationships between the three factors. Therefore, it is important to recognize that PIU can have a detrimental effect on mental and physical health as well as on oral health. Based on this study’s results, it is necessary for parents to have adequate control over adolescents’ PIU. It is also important to improve awareness of Internet addiction and consider counseling and appropriate interventions for potential and high-risk groups through well-designed health promotion programs to prevent internet addiction in adolescents.

## Figures and Tables

**Table 1:** Oral disease symptoms experienced (number of participants with percentage in parentheses) according to general characteristics (*N* = 73,238).

Variable	Category	Oral Disease Symptoms *N* (Weighted %)		*p*-Value
		Yes	No	
Sex	Boys	23,973 (61.9)	14,418 (38.1)	<0.001
	Girls	24,551 (70.2)	10,296 (29.8)	
Grade	Middle School 1st (13-years-old)	7557 (60.2)	4911 (39.8)	<0.001
	Middle school 2nd (14-years-old)	7559 (61.4)	4740 (38.6)	
	Middle school 3rd (15-years-old)	8183 (64.6)	4420 (35.4)	
	High school 1st (16-years-old)	8438 (69.4)	3583 (30.6)	
	High school 2nd (17-years-old)	8440 (68.9)	3684 (31.1)	
	High school 3rd (18-years-old)	8147 (70.4)	3376 (29.6)	
Academic Performance	High	17,036 (65.1)	8915 (34.9)	<0.001
	Middle	12,795 (64.4)	6856 (35.6)	
	Low	18,693 (67.5)	8943 (32.5)	
Perceived Family Economic Status	High	12,637 (59.7)	8435 (40.3)	<0.001
	Middle	22,558 (65.5)	11,695 (34.5)	
	Low	13,329 (74.1)	4584 (25.9)	
Living Status	Living with family	45,885 (65.8)	23,457 (34.2)	<0.001
	Living with relatives	765 (67.9)	355 (32.1)	
	Boarding, living in dorm	1600 (67.4)	665 (32.6)	
	Living in a care facility	274 (53.0)	237 (47.0)	

Data analysis used Rao-Scott χ^2^-test. *p* < 0.05.

**Table 2:** Oral disease symptoms experienced (number of participants, with percentage in parentheses) according to health-related factors and problematic Internet use.

Variable	Category	Oral Disease Symptoms *N* = 74,238 (Weighted %)		*p*-Value
		Yes	No	
**Health Related Factors**				
Alcohol	Yes	28,240 (69.6)	12,118 (30.4)	<0.001
	No	20,284 (61.2)	12,596 (38.8)	
Smoking	Yes	13,738 (70.5)	5647 (29.5)	<0.001
	No	34,786 (64.2)	19,067 (35.8)	
Stress Level	Not at all	725 (42.0)	969 (58.0)	<0.001
	Not much	5144 (51.4)	4367 (48.6)	
	A little	19,022 (63.8)	10,647 (36.2)	
	A lot	16,427 (72.5)	6158 (27.5)	
	Very much	7206 (75.3)	2303 (24.7)	
Sleep Satisfaction	Completely sufficient	3073 (55.2)	2438 (44.8)	<0.001
	Sufficient	8866 (59.4)	5950 (40.6)	
	Moderate	16,123 (64.9)	8443 (35.1)	
	Not sufficient	13,726 (70.7)	5471 (29.3)	
	Not at all sufficient	6736 (74.3)	2412 (25.7)	
Sleep Duration (night/per day)	Less than 5 h	5832 (66.4)	2851 (33.6)	0.201
	≥5–7 h	31,738 (65.9)	16,040 (34.1)	
	≥7–9 h	10,382 (65.0)	5528 (35.0)	
	More than 9 h	572 (65.9)	295 (34.1)	
^a^ PIU Group	General group	45,340 (65.0)	23,857 (35.0)	<0.001
	Potential risk group	1468 (78.8)	398 (21.2)	
	High risk group	1716 (79.9)	459 (20.1)	
Time of Internet Use (hours/day, weekend)	Less than 1 h	1408 (66.7)	699 (33.3)	<0.001
	≥1–2 h	7859 (63.3)	4478 (36.7)	
	≥2–3 h	10,279 (66.5)	5162 (33.5)	
	≥3–4 h	7760 (68.5)	3546 (31.5)	
	≥4–5 h	4552 (69.4)	1988 (30.6)	
	≥5–6 h	2661 (73.4)	998 (26.6)	
	More than 6 h	3679 (72.4)	1387 (27.6)	

^a^ PIU: Problematic Internet Use. Data analysis used the Rao-Scott χ^2^ test. *p* < 0.05; Time of Internet use (*N* = 56,456): only respondents who answered “*yes*” to the former question were required to answer the latter.

**Table 3:** Risk of oral disease symptoms according to problematic Internet use and sleep problems by gender.

Variable	Model I ^a^ OR (95% CI)	Model II ^b^ AOR (95% CI)^d^	Model III ^c^ AOR (95% CI)
**Problematic Internet Use**			
**Group (Boys)**			
General group	1	1	1
Potential risk group	1.78 (1.49–2.14)	1.74 (1.45–2.09)	1.59 (1.32–1.91)
High risk group	2.25 (1.91–2.64)	2.14 (1.82–2.53)	1.92 (1.63–2.28)
**Duration of Internet use** **(h/day, weekends)**			
Within 1 h	1	1	1
≥1–2 h	0.85 (0.71–1.02)	0.85 (0.70–1.02)	0.86 (0.71–1.03)
≥2–3 h	0.98 (0.82–1.17)	0.97 (0.81–1.17)	0.96 (0.80–1.15)
≥3–4 h	1.05 (0.87–1.27)	1.04 (0.86–1.27)	1.01 (0.84–1.23)
≥4–5 h	1.04 (0.85–1.27)	1.02 (0.82–1.26)	0.96 (0.78–1.19)
≥5–6 h	1.24 (1.01–1.53)	1.23 (0.99–1.51)	1.13 (0.92–1.41)
More than 6 h	1.16 (0.95–1.42)	1.12 (0.92–1.38)	1.03 (0.84–1.26)
**Sleep satisfaction**			
Completely sufficient		1	1
Sufficient		1.15 (1.03–1.28)	1.04 (0.93–1.16)
Moderately sufficient		1.45 (1.31–1.60)	1.19 (1.07–1.31)
Not sufficient		1.87 (1.66–2.11)	1.41 (1.25–1.59)
Not at all sufficient		2.01 (1.76–2.29)	1.40 (1.23–1.60)
**Sleep duration**		0.99 (0.97–1.02)	1.01 (0.97–1.04)
**Problematic Internet Use Group (Girls)**			
General group	1	1	1
Potential risk group	2.27 (1.74–2.97)	2.15 (1.65–2.80)	1.89 (1.44–2.48)
High risk group	2.41 (1.83–3.17)	2.23 (1.70–2.93)	1.98 (1.50–2.63)
**Duration of Internet use** **(h/day, weekends)**			
Within 1 h	1	1	1
≥1–2 h	0.90 (0.79–1.04)	0.90 (0.78–1.04)	0.89 (0.77–1.02)
≥2–3 h	1.05 (0.90–1.22)	1.04 (0.90–1.20)	0.96 (0.83–1.11)
≥3–4 h	1.19 (1.02–1.40)	1.17 (0.99–1.37)	1.04 (0.89–1.23)
≥4–5 h	1.35 (1.14–1.59)	1.29 (1.09–1.53)	1.12 (0.94–1.32)
≥5–6 h	1.69 (1.40–2.04)	1.61 (1.33–1.94)	1.38 (1.14–1.67)
More than 6 h	1.48 (1.23–1.77)	1.40 (1.16–1.67)	1.17 (0.97–1.40)
**Sleep satisfaction**			
Completely sufficient		1	1
Sufficient		1.06 (0.92–1.22)	1.00 (0.87–1.16)
Moderately sufficient		1.26 (1.09–1.46)	1.07 (0.93–1.24)
Not sufficient		1.59 (1.38–1.83)	1.23 (1.07–1.42)
Not at all sufficient		2.10 (1.81–2.44)	1.44 (1.24–1.67)
Sleep duration		0.99 (0.97–1.02)	1.01 (0.99–1.04)

^a^ Model I: Unadjusted odds ratio (OR) (95% confidence interval (CI)); ^b^ Model II: Adjusted for perceived sleep satisfaction, sleep duration; ^c^ Model III: Adjusted for all covariates (gender, grade, perceived academic performance, perceived family economic status, living status, alcohol, smoking, perceived stress level); ^d^ AOR (95% CI): Adjusted Odds Ratio (95% CI)

**Table 4:** Subgroup analysis of the risk of each 6 oral disease symptoms according to problematic Internet use group.

Variable (Six Oral Disease Symptoms)	PIU Group	Model I OR (95% CI)	Model II ^a^ AOR (95% CI)
Chipped or broken tooth (Yes)	General group	1	1
	Potential group	1.29 (1.116–1.487)	1.03 (0.872–1.211)
	High risk group	1.60 (1.392–1.829)	1.14 (0.977–1.334)
Toothache when eating	General group	1	1
	Potential group	1.77 (1.590–1.971)	1.53 (1.357–1.856)
	High risk group	1.88 (1.702–2.083)	1.71 (1.523–1.923)
Throbbing and sore tooth	General group	1	1
	Potential group	1.66 (1.48–1.875)	1.55 (1.349–1.786)
	High risk group	1.96 (1.770–2.164)	1.71 (1.520–1.927)
Sore and bleeding gums	General group	1	1
	Potential group	1.65 (1.466–1.853)	1.40 (1.215–1.601)
	High risk group	2.11 (1.905–2.331)	1.63 (1.458–1.827)
Pain in tongue or inside Cheeks	General group	1	1
	Potential group	1.72 (1.501–1.969)	1.49 (1.265–1.756)
	High risk group	2.29 (2.052–2.548)	1.90 (1.667–2.164)
Bad breath	General group	1	1
	Potential group	2.05 (1.838–2.290)	1.69 (1.497–1.900)
	High risk group	2.66 (2.407–2.930)	2.05 (1.825–2.298)

^a^ Model II: Adjusted for sex, grade, academic performance, perceived family economic status, living status, alcohol. smoking, stress level, sleep satisfaction, sleep duration, time of internet use; All six oral disease symptoms reference group: “NO”.

## References

[1] Heo J., Oh J., Subramanian S.V., Kim Y., Kawachi I. (2014). Addictive internet use among Korean adolescents: A national survey. PLoS ONE.

[2] Kim Y., Park J.Y., Kim S.B., Jung I.K., Lim Y.S., Kim J.H. (2010). The effects of Internet addiction on the lifestyle and dietary behavior of Korean adolescents. Nutr. Res. Pract..

[3] Park S. (2014). Associations of physical activity with sleep satisfaction, perceived stress, and problematic Internet use in Korean adolescents. BMC Public Health.

[4] Park M.H., Jeon H.O. (2013). Relationships between health behaviors, mental health and internet addiction by gender differences among Korean adolescents. J. Korean Acad. Ind. Coop. Soc..

[5] Blinka L., Skarupova K., Sevcikova A., Wolfling K., Muller K.W., Dreier M. (2015). Excessive Internet use in European adolescents: What determines differences in severity?. Int. J. Public Health.

[6] Nuutinen T., Roos E., Ray C., Villberg J., Valimaa R., Rasmussen M., Holstein B., Godeau E., Beck F., Léger D. (2014). Computer use, sleep duration and health symptoms: A cross-sectional study of 15-year-olds in three countries. Int. J. Public Health.

[7] Park S.J., Ko K.D., Shin S.I., Ha Y.J., Kim G.Y., Kim H.A. (2014). Association of oral health behaviors and status with depression: Results from the Korean National Health and Nutrition Examination Survey, 2010. J. Public Health Dent..

[8] Kaess M., Durkee T., Brunner R., Carli V., Parzer P., Wasserman C., Sarchiapone M., Hoven C., Apter A., Balazs J. (2014). Pathological Internet use among European adolescents: Psychopathology and self-destructive behaviours. Eur. Child Adolesc. Psychiatry.

[9] Kelley K.J., Gruber E.M. (2013). Problematic Internet use and physical health. J. Behav. Addict..

[10] Lam L.T. (2014). Risk factors of Internet addiction and the health effect of internet addiction on adolescents: A systematic review of longitudinal and prospective studies. Curr. Psychiatry Rep..

[11] An J., Sun Y., Wan Y., Chen J., Wang X., Tao F. (2014). Associations between problematic internet use and adolescents’ physical and psychological symptoms: Possible role of sleep quality. J. Addict. Med..

[12] Lam L.T. (2014). Internet gaming addiction, problematic use of the internet, and sleep problems: A systematic review. Curr. Psychiatry Rep..

[13] Cain N., Gradisar M. (2010). Electronic media use and sleep in school-aged children and adolescents: A review. Sleep Med..

[14] Chen Y.L., Gau S.S.-F. (2016). Sleep problems and internet addiction among children and adolescents: A longitudinal study. J. Sleep Res..

[15] Christensen L., Somers S. (1996). Comparison of nutrient intake among depressed and nondepressed individuals. Int. J. Eat. Disord..

[16] Choi K., Son H., Park M., Han J., Kim K., Lee B., Gwak H. (2009). Internet overuse and excessive daytime sleepiness in adolescents. Psychiatry Clin. Neurosci..

[17] Grover V., Malhotra R., Kaur H. (2015). Exploring association between sleep deprivation and chronic periodontitis: A pilot study. J. Indian Soc. Periodontol..

[18] Wiener R.C. (2016). Relationship of routine inadequate sleep duration and periodontitis in a nationally representative sample. Sleep Disord..

[19] Besedovsky L., Lange T., Born J. (2012). Sleep and immune function. Pflug. Arch..

[20] Dinges D.F., Douglas S.D., Hamarman S., Zaugg L., Kapoor S. (1995). Sleep deprivation and human immune function. Adv. Neuroimmunol..

[21] Carra M.C., Schmitt A., Thomas F., Danchin N., Pannier B., Bouchard P. (2017). Sleep disorders and oral health: A cross-sectional study. Clin. Oral Investig..

[22] Celik C.B., Odaci H., Bayraktar N. (2015). Is problematic internet use an indicator of eating disorders among Turkish university students?. Eat. Weight Disord..

[23] Do K.Y., Lee E.S., Lee K.S. (2017). Association between excessive internet use and oral health behaviors of Korean adolescents: A 2015 national survey. Community Dent. Health.

[24] World Health Organization (2012). Oral Health Fact Sheet No. 318. http://www.who.int/oral_health/publications/factsheet/en/.

[25] Soares Luis H.P., Assunçao V.A., Soares Luis L.F. (2016). Oral health habits, attitudes and behaviors of Portuguese adolescents. Int. J. Adolesc. Med. Health.

[26] Calderon S.J., Mallory C. (2014). A systematic review of oral health behavior research in American adolescents. J. Sch. Nurs..

[27] Lam L.T. (2015). Parental mental health and Internet Addiction in adolescents. Addict. Behav..

[28] Park S., Hong K.E., Park E.J., Ha K.S., Yoo H.J. (2013). The association between problematic internet use and depression, suicidal ideation and bipolar disorder symptoms in Korean adolescents. Aust. N. Z. J. Psychiatry.

[29] Reed P., Vile R., Osborne L.A., Romano M., Truzoli R. (2015). Problematic Internet Usage and Immune Function. PLoS ONE.

[30] Yoo Y.S., Cho O.H., Cha K.S. (2014). Associations between overuse of the internet and mental health in adolescents. Nurs. Health Sci..

[31] Ministry of Science and ICT, National Information Society Agency (2017). The Survey on Smart Phone over Dependence.

[32] Reners M., Brecx M. (2007). Stress and periodontal disease. Int. J. Dent. Hyg..

[33] Kim B.-K. (2008). The Follow up Study of Internet Addiction Proneness Scale.

[34] Chen M., Anderson R.M., Barmes D.E., Leclercq M.-H., Lyttle C.S. (1997). Comparing Oral Health Care Systems: A Second International Collaborative Study.

[35] Korea Internet and Security Agency (2014). Survey of Internet Usage.

[36] Block J.J. (2008). Issues for DSM-V: Internet addiction. Am. J. Psychiatry.

[37] American Psychiatric Association (2013). Diagnostic and Statistical Manual of Mental Disorders (DSM-5).

[38] Griffiths M.D., Kuss D.J., Billieux J., Pontes H.M. (2016). The evolution of Internet addiction: A global perspective. Addict. Behav..

[39] Wu C.Y., Lee M.B., Liao S.C., Chang L.R. (2015). Risk factors of internet addiction among Internet users: An online questionnaire survey. PLoS ONE.

[40] Sundararajan S., Muthukumar S., Rao S.R. (2015). Relationship between depression and chronic periodontitis. J. Indian Soc. Periodontol..

[41] Tothova L., Celecova V., Celec P. (2013). Salivary markers of oxidative stress and their relation to periodontal and dental status in children. Dis. Markers.

[42] Werner H., Hakeberg M., Dahlstrom L., Eriksson M., Sjogren P., Strandell A., Svanberg T., Svensson L., Wide Boman U. (2016). Psychological Interventions for poor oral health: A systematic review. J. Dent. Res..

[43] Korea Centers for Disease Control and Prevention (2011). Korea Youth Risk Behavior Web-Based Survey 2010: Survey Press Releases.

[44] Johannsen A., Rylander G., Soder B., Asberg M. (2006). Dental plaque, gingival inflammation, and elevated levels of interleukin-6 and cortisol in gingival crevicular fluid from women with stress-related depression and exhaustion. J. Periodontol..

[45] Rosania A.E., Low K.G., McCormick C.M., Rosania D.A. (2009). Stress, depression, cortisol, and periodontal disease. J. Periodontol..

[46] Irwin M. (2002). Effects of sleep and sleep loss on immunity and cytokines. Brain Behav. Immun..

[47] Acar M., Türkcan İ., Özdaş T., Bal C., Cingi C. (2015). Obstructive sleep apnoea syndrome does not negatively affect oral and dental health. J. Laryngol. Otol..

[48] Majde J.A., Krueger J.M. (2005). Links between the innate immune system and sleep. J. Allergy Clin. Immunol..

[49] Park H. (2015). The relationship between Internet use for non-academic purposes and happiness and stress in adolescents. J. Korean Acad. Community Health Nurs..

[50] Romano M., Osborne L.A., Truzoli R., Reed P. (2013). Differential Psychological Impact of Internet Exposure on Internet Addicts. PLoS ONE.

